# Infantile Paralysis

**Published:** 1867-02

**Authors:** Chas. F. Taylor

**Affiliations:** N.Y.


### ARTICLE XII.

To the Editor of the Chicago Medical Examiner:—

Sir:—I wish to call your attention, and through the pages of your excellent Journal, that also of the profession generally, to a little work lately issued from the press of J. B. Lippincott & Co., Philadelphia, and entitled "Infantile Paralysis, and its attendant Deformities."

The increasing frequency of this malady in this country, especially among the more active and intelligent portion of the community, and the meagreness of literature on this really important subject is, it would seem, a sufficient reason why every physician should welcome the appearance of this really practical, and, therefore, valuable little treatise. It is the production of Dr. Charles F. Taylor, of New York City, who is well known to the profession as an earnest and successful worker in the department of orthopædic surgery, and whose wide experience in the treatment of this class of cases has eminently qualified him for his task.

This monograph is characterized by the same originality of thought and sound common sense which have distinguished the previous literary efforts of this writer. The ideas therein presented are not to be looked upon as the idle speculations of the theorist, but may be safely accepted as facts which have been established upon the firm foundation of extensive and prolonged observation, and which may be verified by any physician in whose practice cases of infantile paralysis may occur.

After defining the term "Infantile Paralysis," and briefly glancing at its causes, the increasing frequency of its occurrence, its pathology and symptomatology, and mentioning some of the peculiar characteristics of this form of paralysis, the author passes on to the treatment, the consideration of which occupies the larger portion of the work. Considerable space is devoted to the examination of the mechanical causes of the various distortions, which are likely to, and, indeed, almost always do, occur during the progress of this malady, but which the writer clearly demonstrates to be entirely preventable.

The facts and arguments contained in the pages of this interesting and original little book are finally summed up in the following propositions, viz.:—

"1. Infantile paralysis is an arrest of vegetative development from some unknown cause.

"2. The characteristics of this form of paralysis suggest a peripheric blight rather than a loss of central nerve power.

"3. With diminished nutrition, temperature, and muscular power, there is also diminished muscular irritability; and there is no such thing as involuntary or reflex contraction in infantile paralysis.

"4. The shortening of certain muscles, is not a necessary consequence of infantile paralysis; and when it does occur, it is simply the adaptation of their length to the position they happen to be in.

"5. It is entirely accidental *which* muscles become shortened, whether flexors or extensors.

"6. Hence, deformities are not a necessary consequence of infantile paralysis, and when they are allowed to occur, the process of recovery is arrested.

"7. When deformities have already formed, they should be treated for an ultimate end, viz.: to bring the patient back to the place from which he should not have been allowed to diverge, where the treatment for his *paralysis* should begin.

"8. Hence, tenotomy and mechanical appliances are only means to an end—the first steps of a course of treatment having in view the restoration of the muscular power.

"9. The most natural means for this purpose is the supply of local heat—involving increased local circulation—together with local exercise, corresponding with the position and ability of the part exercised.

"10. The element of *time* must also be taken into consideration."

The foregoing views are elucidated by the relation of some eight or ten cases, the histories of which are well given, and fully illustrate the points which the writer makes.

Not the least interesting feature of the work is to be found in the beautiful wood-cuts which adorn its pages, giving the reader a very clear idea of the distortions usually following the accession of this form of paralysis, and also completely illustrating that form of treatment which the author has found to be followed by the best results in these cases. E.

